# Metabolic Control in Mammalian Fed-Batch Cell Cultures for Reduced Lactic Acid Accumulation and Improved Process Robustness

**DOI:** 10.3390/bioengineering3010005

**Published:** 2016-01-11

**Authors:** Viktor Konakovsky, Christoph Clemens, Markus Michael Müller, Jan Bechmann, Martina Berger, Stefan Schlatter, Christoph Herwig

**Affiliations:** 1 Institute of Chemical Engineering, Division of Biochemical Engineering, Vienna University of Technology, Gumpendorfer Strasse 1A 166-4, 1060 Vienna, Austria; vkonakovtuwien@gmail.com; 2 Boehringer Ingelheim Pharma GmbH & Co. KG Dep. Bioprocess Development, Biberach, Germany; christoph.clemens@boehringer-ingelheim.com (C.C.); markus_michael.mueller@boehringer-ingelheim.com (M.M.M.); jan.bechmann@boehringer-ingelheim.com (J.B.); martina.berger@boehringer-ingelheim.com (M.B.); stefan.schlatter@boehringer-ingelheim.com (S.S.)

**Keywords:** CHO cell culture, scale-down, fed batch, automation, Lactic acid control, pH, metabolic control, MVDA, uncertainty, online analyzer

## Abstract

Biomass and cell-specific metabolic rates usually change dynamically over time, making the “feed according to need” strategy difficult to realize in a commercial fed-batch process. We here demonstrate a novel feeding strategy which is designed to hold a particular metabolic state in a fed-batch process by adaptive feeding in real time. The feed rate is calculated with a transferable biomass model based on capacitance, which changes the nutrient flow stoichiometrically in real time. A limited glucose environment was used to confine the cell in a particular metabolic state. In order to cope with uncertainty, two strategies were tested to change the adaptive feed rate and prevent starvation while in limitation: (i) inline pH and online glucose concentration measurement or (ii) inline pH alone, which was shown to be sufficient for the problem statement. In this contribution, we achieved *metabolic control* within a defined target range. The direct benefit was two-fold: the lactic acid profile was improved and pH could be kept stable. Multivariate Data Analysis (MVDA) has shown that pH influenced lactic acid production or consumption in historical data sets. We demonstrate that a low pH (around 6.8) is not required for our strategy, as glucose availability is already limiting the flux. On the contrary, we boosted glycolytic flux in glucose limitation by setting the pH to 7.4. This new approach led to a yield of lactic acid/glucose (Y L/G) around zero for the whole process time and high titers in our labs. We hypothesize that a higher carbon flux, resulting from a higher pH, may lead to more cells which produce more product. The relevance of this work aims at feeding mammalian cell cultures safely in limitation with a desired metabolic flux range. This resulted in extremely stable, low glucose levels, very robust pH profiles without acid/base interventions and a metabolic state in which lactic acid was consumed instead of being produced from day 1. With this contribution, we wish to extend the basic repertoire of available process control strategies, which will open up new avenues in automation technology and radically improve process robustness in both process development and manufacturing.

## 1. Introduction

### 1.1. Problem Statement

A great amount of modern biopharmaceuticals such as monoclonal Antibodies (mAbs), fusion proteins, bi-specific antibodies, IgG and others are produced today by highly specialized cells in a fed-batch bioprocess. The Chinese hamster ovary (CHO), well known by cell banking institutes and authorities, is often engineered (*i.e.*, GS-CHO) to satisfy a tailored production purpose. Often a large-scale fed-batch or perfusion operation is required to meet the market demand. In such processes, the feeding regime was found to have a great impact on lactate production, which negatively affects the productivity [1]. As a consequence, variations in both quantity and quality during recombinant protein processes affect the profitability of the production plant markedly.

The lactic acid profile of a mammalian cell culture process affects the maximum achievable cell count and final product titer [2,3]. Manufacturing runs with a high and a low lactic acid profile have been linked with productivity of the process by using Multivariate Data Analysis (MVDA) methods [4,5]. It does not come as a big surprise that there is not only one but there are several approaches to decrease lactic acid. Some methods encompass genetic modifications of the clone [6,7,8], changes in medium composition [9,10,11,12], interventions on a genetic level [13,14], feeding strategy [15,16,17,18], modification of metabolic state [19,20,21,22,23] or the physico-chemical environment [24,25,26,27,28,29,30], to name a few.

As lactic acid is produced, pH falls—which can again impair cell proliferation [31] at critically low pH. Depending on the clone and cell type, the lower critical pH limit is typically between 6.6–6.8 in mammalian cell culture [24]. In several other studies, pH was also associated with interfering with product quality [32]; therefore, the way pH is controlled is still an interesting question today. Ideally, pH should be fixed during the whole process and should never need adjustment by either acidic or alkaline control agents. In practice this is not exactly the case. An increase of osmolality and stress to the cells is often the result of a suboptimal pH control strategy. Operation at low pH is observed to restrict the formation of lactic acid at the cost of inhibiting cellular proliferation while an alkaline pH has the opposite effect [24,25,33]. Therefore, many industrial processes start with high pH and shift to low pH during the culture as will be seen a bit later in this contribution. However, modifying the pH alone is not fast enough to prevent lactic acid build-up, as can be seen in the lactic acid profiles in our data—the metabolic state itself must be modified, and this can be done with an appropriate feeding strategy.

### 1.2. Feeding Strategies

The choice of an appropriate feeding strategy depends very much on the context; they may be based on well-defined industrial production runs, where little variation is anticipated and a historical run can be used as template for the next run. Alternatively, they are derived by in-line, at-line or on-line signals from the current process in real time, which is often the case in a bioprocess development environment [34,35,36]. Therefore, a methodology to deliver feed adaptively, accurately and safely in mammalian cell culture development is of very high relevance [37,38,39,40]. In order to control lactate production by adaptive feeding, the main understanding is that a limited metabolic state, mainly a carbon limitation, needs to be designed. The methodology is typically divided into data-driven and adaptive feeding approaches.

#### 1.2.1. Data-Driven Feeding

Data driven approaches rely on knowledge gathered from historical data. Drapeau [16], Luan [15,41] and Gagnon *et al.* [42] published methods to control mammalian cell culture in glucose limitation (around 1 mM). As criteria for an increase or decrease of a previously determined feed rate from very similar historical runs, if too much lactic acid is produced, the feed is too high and needs to decrease, whereas when the pH is rising, the feed is too low and needs to increase. This intuitive and simple method may be limited if buffers are employed, which mask lactic acid production and may lead to periods of starvation [43]. Aehle *et al.* [44] described the control of a mammalian fed-batch cultivation by limiting glutamine instead of glucose availability, thus affecting carbon source utilization and reducing ammonia yields, to control the feed rate. Previous experiments under similar conditions or numerical optimization studies were used to establish a tcOUR (total cumulative oxygen uptake rate) profile. This profile was correlated *ab initio* to the historical viable biomass profile. Future repetitions of the experiment with different initial viable biomass concentrations led to a highly reproducible cell concentration estimation and also to the desired sub-maximal specific growth rate. In order to deliver the correct amount of feed in real time to the variable biomass, the glutamine per biomass yield (Y X/GLN) had to be known and proved to be fairly constant during the six days of cultivation.

Liu *et al.* [45] even suggested the feed-forward control of mammalian cell cultures. They derived a historical growth rate by linear calibration of previous growth curves and used it together with the yield of biomass per glucose (Y X/GLC) to set up a determined feed rate. Validation with three different cell lines led to an improvement of titer as well as IVCC (integrated viable cell concentration). This leads to the question of why data-driven strategies work so extremely well. One reason is that the validation experiments are short so that the verification does not face the difficulty of describing a considerable death phase, which leads to a reduced representation of a typical industrial process. Additionally, different clones in different scales often have different biomass and metabolite profiles. Differences in medium composition significantly diminish the usefulness of pre-determined feed profiles. Typically, even large datasets of one clone in scale 1 cannot predict the behavior of another clone in scale 1 or the same clone in scale 2. Therefore, a sufficient number of experiments are typically conducted *a priori*, which can build up enough confidence that a data-driven approach does not fail, which would be especially devastating in a manufacturing-scale run.

#### 1.2.2. Adaptive Feeding Strategies

Usual orthogonal process development runs are too different to successfully apply a data-driven pre-calculated feed or biomass profile from historical data. For these reasons we need to focus on simple mechanistic relationships which allow automatic feed rate adaptation to the current and not a past process. A look into literature shows that there are several ways to accomplish this by deriving a feed in real time using in-line, at-line and on-line signals.

Zhou and Hu first mention signals which may be used to detect the metabolic state of mammalian cell cultures and derive a feeding profile on-line [46]. The turbidity probe allowed the conversion of optical density (OD) to cell concentration with a simple calibration curve as well as the oxygen uptake rate (OUR) which made it possible to detect a more direct measure for cellular activity. Noll and Biselli [47] used a capacitance probe to deliver feed to a fluidized bed culture. The yield X/GLN was constant during the whole time which allowed keeping glutamine at 0.45 mM with an online massflow dosing system. Dowd *et al.* [48] used capacitance as well to estimate viable cell concentrations in a perfusion process to control the nutrient feed rate. The feeding was adjusted automatically and was used to maximize the productivity. Zhou *et al.* [49] used the OUR measurement to adjust the nutrient feeding rate in a fed-batch culture. This was possible by applying a previously determined yield of glucose per oxygen (GLC/OX) consumption and led to the control of the process at very low glucose and amino acid levels. The strategy was applicable as long as a reliable calculation of the kLa (volumetric oxygen transfer coefficient) allowed determination of OUR. Europa *et al.* [50] used such a setup to show how controlling substrates at low levels not only led to reduced metabolite formation but also to a multiplicity of steady states, in which a more efficient metabolism was observed. Ozturk *et al.* [38] utilized an at-line analyzer to manipulate the perfusion rate of a culture in real time. Li *et al.* [51] proposed several other techniques to control mammalian cell culture, which are based on the cell’s specific oxygen uptake rate (qOUR) during the process. In combination with substrate or metabolite measurements, qOUR was used as an indicator to change other process parameters on-line. Among those, feed rate, pH, temperature, stirring speed, and pO_2_ were adapted when limitations, such as reduced qOUR or low substrate, could be detected. Lu [37] compared two methods to control the feed rate: in one instance, an auto sampler method was used to hold a particular glucose concentration, and in the other, a capacitance signal was used as a surrogate for cell growth to control the feed rate. In both cases, the target glucose concentration was non-limited between 4–6 g/L to prevent over- and under-feeding. Variable, specific consumption rates were observed over time, which are typical for operations at such high concentration ranges of substrate. As an online or offline measurement was available, it could be used to correct these deviations. Alternatively, previous experiments were shown to be useful to deduct the evolution of the specific consumption rates, with the drawback that this again makes the process dependent on historical data.

### 1.3. Challenges in Process Control

The aforementioned contributions describe the state of the art; however, there are still many gaps in knowledge which need to first be mentioned and then attempted to be closed. Many methods are based on some prior knowledge of the process. However, in reality there are many sources for deviations which complicate things considerably. Some sources of complication with which we were confronted in our labs and want to report are listed in Table 1.

**Table 1:** Selection of observed sources of complications in the development of feeding strategies in our labs.

Source	Influence
Clones	Metabolic needs may differ greatly, leading to the perpetual development of historical feeding profiles. In adaptive feeding regimes, clone-dependent differences of dielectric properties may complicate biomass estimation when capacitance probes are used, while turbidity probes may detect more or less cell debris in the decline phase, depending on which clone was used.
Scales	Especially on-line offgas/kLa–dependent control strategies may become very difficult to transfer because they depend on the aeration and stirrer cascade strategy (*i.e.*, constant or adaptively increasing gas flow to hold pO_2_).
Assumptions	Constant yields (*i.e.*, GLC/OX, X/GLC, X/GLN, *etc.*) may change over time, leading to stoichiometric over- or under-feeding.
Media	Addition of growth-influencing components may change historical feeding profiles completely and make a direct comparison between experiments difficult as these changes have further implications on the process.
Process parameters	Changes in temperature, stirrer speed, pO_2_, pH or pCO_2_ levels may affect gas solubility, buffer capacity, offgas profiles, cellular stress level, and growth and may change the metabolic requirements for both adaptively or historically calculated feed rates.

We do not claim to offer solutions to all mentioned points but want to make the reader aware of their existence and propose solutions for some of them in our own work: the herewith presented adaptive feeding strategy was based on estimation of biomass in the current process, making it independent of historical data. The method we used in our labs to estimate biomass, and with it the adaptive feeding rate, has been shown in a previous contribution to be transferrable between clones and scales [52].

We coped with changing metabolic yields by limiting glucose concentrations and the glucose uptake rate to a desired range. The yield was still subject to variation, but could not exceed a particular range—the rate at which substrate was supplied corresponded to the desired consumption rate of the cells in real time, which made it controllable. The method is based on a first principle investigation using correlations between the specific rates such as glucose or lactic acid to determine the feed rate set-point. The metabolic response may differ from clone to clone in the slope of the yield Y L/G, but once established, this correlation will hold for one clone which makes the general methodology applicable to different clones. Uncertainty consisting of both metabolic state and biomass was considered by applying a range for the feed set-point; even though the yields and biomass error are subject to change, certain signals (*i.e.*, pH, among others) could be used to switch the feeding rate automatically to ensure sufficient supply of substrate for the whole process time.

### 1.4. Goal

The goal of this study was two-fold: to reduce lactic acid levels and to minimize the pH control actions by additional acid-base feeding using a dynamic process environment as encountered in a process of industrial relevance. This contribution therefore proposes a novel adaptive feeding strategy, which is based on estimating the viable cell concentration with a capacitance probe in glucose-limited growth conditions, targeting a low lactic acid/glucose yield (Y L/G) and taking pH variation into account in the control strategy. The workflow followed in this contribution was (i) to analyze historical process development data to get an understanding in which range specific glucose consumption shows a favorable Y L/G profile; (ii) MVDA to determine the contributions of important parameters influencing lactic acid production; and (iii) development of the feeding strategy based on a high and low uncertainty of the metabolic state and biomass estimation error. The results show that lactic acid build-up can be decreased by confining glucose flux to a particularly low range, regardless of the pH set-point. However, pH may have helped to keep glucose consumption higher than reference processes late in the process, which in turn may have had a positive effect on productivity. The challenge was finding a way to address this in a dynamic fed-batch process; therefore, our methodology aims at being directly transferrable from process development back to manufacturing conditions.

## 2. Experimental Section

### 2.1. Cell Lines

All data in this contribution was collected from one single engineered CHO clone (derived from CHO DG44), subsequently referred to as “Clone B”. This clone was kindly provided by Boehringer Ingelheim (Ingelheim, Germany) for the necessary experiments at the VUT (Vienna University of Technology). Only Clone B data was used for data analysis purposes as the metabolic behavior is different from Clone A data. However, for completeness, the data to construct and validate the multivariate model for biomass estimation (see chapter MVDA) were recorded with “Clone A” clones. The biomass model was shown to be transferrable and also estimate Clone B data. Clone A data is not discussed further in this contribution, but more information could be found in our recent publication [52].

### 2.2. Available Dataset

A historical Clone B dataset consisting of 29 fed-batch fermentations from process development (2L and 80L) was kindly provided by Boehringer Ingelheim (Ingelheim, Germany) to generate process understanding using MVDA techniques. These data sets are also referred to as “Historical data”. On this basis, fed-batch fermentations were mirrored in a scale-down model (2L), and two experiments, “R-30” and “R-31”, were selected and described in detail in this contribution.

### 2.3. Media

Media for the seed train and fed-batches are proprietary in composition and subject to variations in starting levels of metabolites, growth factors, selection pressure, *etc.* All components were serum-free, animal-component-free, and chemically defined.

### 2.4. Process Setup

Clone B cells were cultivated in shake-flasks (Corning Inc., Corning, NY, USA) in incubators (Minitron, Infors, Bottmingen, Switzerland) with 5% partial CO_2_ pressure at physiological temperature (35–37 °C) on orbital shakers at 120 rpm (orbit 50 mm). Passaging was performed every third day in proprietary chemically defined media and the bioreactors (3.6L, Infors HT, Bottmingen, Switzerland) were inoculated at an initial seed density of 3 × 10^4^ to more than 1 × 10^6^ cells/mL. Process information was logged using the process management system Lucullus (PIMS, Lucullus, Biospectra, Switzerland). A capacitance probe (Biomass Sensor, Hamilton Bonaduz AG, Bonaduz, GR, Switzerland) in scanning mode was used with an excitation frequency ranging from 0.3 MHz to 10 MHz, the signal was collected in real time and recorded between one and more than 60 min steps. Offline concentration of total cells, viable cells and viability was measured using an image-based white/dark classification algorithm after automatic trypan blue staining integrated in the cell counter (Cedex HiRes, Roche, Basel, Switzerland). Main metabolite concentrations (Glucose, Lactic acid and IgG) were measured on-line and off-line using a photometric robot (CubianXC, Optocell, Bielefeld, Germany).

### 2.5. Online Enzymatic Analyzer

An enzymatic analyzer (CubianXC, Optocell, Germany) was coupled to the process by withdrawing sample via a ceramic membrane made of Al_2_O_3_ (pore size 0.2 µm, membrane area 17.8 cm^2^, membrane thickness 1.6 mm; IBA, Heiligenstadt, Germany). The supernatant was removed from the reactor at certain intervals at a flow rate between 0.2–1.0 mL/min. The sampling intervals of the analyzer were tested between 3–12 h in experiment R-30 and a designated sampling interval of 6 h was set for the experiment R-31 to measure, among others, glucose and lactic acid concentrations. Purging the lines and sampling took a total of 30 min where a maximum of 12 mL cell-free supernatant was withdrawn per sample. Faster purging and more frequent sampling (up to 30 min intervals) was technically possible, but not required for our purpose. A more detailed description of the enzymatic robot’s set-up and substrate and metabolite measurements is described elsewhere [53].

### 2.6. MVDA

#### 2.6.1. Biomass Model

In brief, multivariate models perform better at capturing the declining phase of a culture than linear models. One of these was available to estimate biomass in historical Clone A runs. The resulting model was transferrable to another clone (here: Clone B) by adaptation of the slope of the multivariate model for this new clone. For more information please refer to our publication [52]. The model was constructed prior to the experiment with Datalab 3.5 software (kindly provided by Prof. Lohninger, Vienna University of Technology, Vienna, Austria) [54] and the transferred Clone A model was used to estimate Clone B biomass in real time.

#### 2.6.2. Data Mining

Historical Clone B data from process development was harmonized by using the same calculation routine in Matlab (Mathworks, Natick, MA, USA) for all runs, including selected experiments R-30 and R-31 at the institute, to exclude possible variations after data treatment. In order to gather process understanding of how and by which importance the process parameters relate to each other, we used PLS-R (partial least squares regression) in Simca 13.0.3 (Umetrics, Sweden) to analyze the historical dataset. These parameters were chosen with considerations explained a little later in the results section and encompassed the specific rates and concentrations of glucose and lactic acid as well as pH, which was used in its non-logarithmic representation of H^+^ concentration.

### 2.7. Process Control

#### 2.7.1. Process Control Scheme

**Figure 1:**
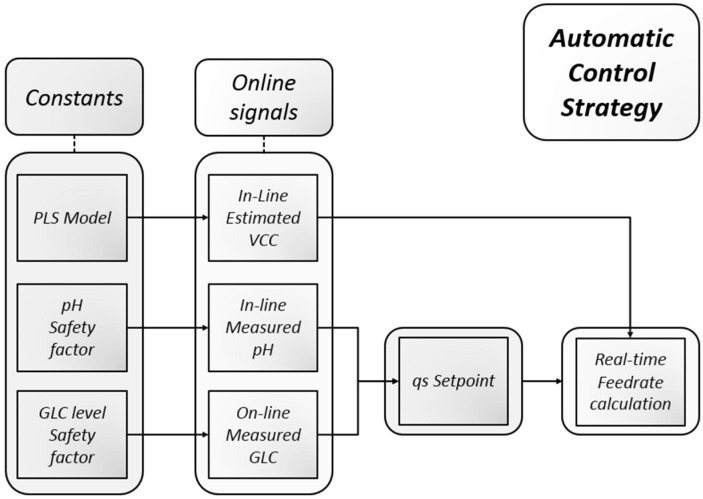
Automatic control strategy using exclusively real-time available signals. Constants (safety factors and the biomass model) were defined *a priori* which led to either a rather loose or very strict adherence to a desired qs set-point.

The recorded spectra were recorded every minute and directly translated into a real-time feed rate. The following script (Figure 1) controlled the process in the experiments. First, biomass was estimated using the coefficients calculated from a multivariate model. We used the assumption that glucose is limited and solely the feed rate is determining the volumetric glucose uptake rate. Both entities are combined to a specific glucose uptake rate, which is in turn used as a set-point to calculate a real-time feed rate. The calculation of specific substrate consumption qs was done by dividing the volumetric rate by the viable cell concentration. As derivative rates tend to be noisy, all specific rates were treated using Matlab’s *rlowess* function [55]. The set-point of the feeding rate was corrected in real time by either of two factors: by an online enzymatic analyzer result (such as threshold glucose concentration), or by pH (for instance a pH upper or lower band), or a combination of both.

#### 2.7.2. Feed Rate Calculation

We estimate biomass and feed stoichiometrically for the specific consumption rate q of a substrate s which the cells should ideally hold. A material balance for substrate glucose was used [56] to calculate qs and historical data analysis was performed to find a useful qs set-point which should be held over time. As qs is not a static value and subject to change, the question of which qs should be set was a very important one. We address it and the implications a bit later in the results section of our work. The feed set-point was controlled gravimetrically by a pump (Lambda Preciflow, Czech Republic) which received the adaptive set-point from a process management system Lucullus (PIMS, Lucullus, Biospectra, Switzerland) in real time. The adaptive feed rate (see Equation (1)) increased and decreased with biomass, which was estimated using a PLS model. The rate at which cells were fed stoichiometrically depended on the experiment and could be influenced by certain process events (Equation (2) and Table 2) to prevent starvation of the cells without having to rely on operator interventions.

The real-time automatic feed rate is described by Equation (1), where qs (which may be glucose or any other substrate) is kept constant.

(1) $$Feed\left[\frac{ml}{h}\right]=\frac{VCC\left[\frac{c}{ml}\right]\cdot V_{Reactor}\left[mL\right]\;\cdot q_{s}\;\left[\frac{mg}{c\cdot h}\right]\;}{Feed\;concentration_{Substrate}\;\left[\frac{mg}{ml}\right]}$$

We used a qGlc *range* instead of a single, fixed set-point, which depends on a previously defined safety factor (ranging here from −25% to +100%). As a switch between qs set-points we used inline pH and online available signals of substrates or metabolites measured by the online analyzer (here glucose); therefore, the qs we specified to be desirable to reduce Y L/G is multiplied by a factor which corresponds to the expected variation in the process. For both experiments, the factor (Equation (2)) by which qs changed was between 0.75 and 2.0 (1.0 minus safety factor of 25% and 1.0 plus safety factor of 100%) and if 1.0 is replaced by the actual feeding set-point, qs reads 8 (rounded up) to 30, depending on the experiment. The safety factor specification by either pH or online analyzer was distinct in both experiments and is explained in detail in Table 2.

(2) $$qs{\;}_{adapted}=qs-\;safety\;factor\;f\left(pH,\;online\;analyzer\right)\;\cdot qs$$

The final equation (Equation (3)) as it was used in the control strategy now reads:

(3) $$Feed\left[\frac{ml}{h}\right]=\frac{VCC\left[\frac{c}{ml}\right]\cdot V_{Reactor}\left[mL\right]\;\cdot q_{s\;adapted}\;\left[\frac{mg}{c\cdot h}\right]\;}{Feed\;concentration_{S,in}\;\left[\frac{mg}{ml}\right]}$$

**Table 2:** Control specifications: Initial qs set-point (SP) in [pg/ch] (picogram per cell per hour), correction by a defined safety factor in [%] under certain conditions (pH and Online Analyzer), and Target SP.

	*Control Specifications*			
	***Switch Conditions***		***Target SP [pg/ch]***	
***Experiment***	***R-30***	***R-31***	***R-30***	***R-31***
**Initialization**	Start feed after 2 h	Start feed after 2 h	−15	−10
**pH control ^†^**				
**pH high**	If pH ≥ 7.1. increase qs by 100%	If pH ≥ 7.4. increase qs by 25%	−30	−13
**pH OK**	If pH between 7.1 and 6.9 use the desired qs	If pH between 7.1 and 6.9 use the desired qs	−15	−10
**pH low**	If pH ≤ 6.9 reduce qs by 25%	If pH ≤ 6.9 reduce qs by 25%	−11	−8
**Online Analyzer control ^‡^**				
**GLC low**	If Gluc ≤ 0.4 increase qs by 100%	Monitoring	−30	−10
**GLC high ^∫^**	If Gluc > 0.4 use the desired qs	Monitoring	−15	−10

**^†^** The pH range was chosen to lie in the physiological range. However, it could be extended to conditions close to the maximum tolerance which may lie somewhere between pH 6.5 and up to 8.0 for mammalian cells [27]; **^‡^** The correction order of the set-point is as follows: first pH, then online analyzer. This is important because if the feed is already reduced by pH, it was not done so a second time by the online analyzer. The online analyzer in experiment R-31 had no purpose other than monitoring the metabolite concentrations; **^∫^** A high glucose concentration is the current status quo in most industrial mammalian cell culture processes.

#### 2.7.3. Operation Window for qs

The set-point for feeding was not fixed but assumed several distinct values in two experiments, which are summarized in Table 2. The rationale for the range calculation of the set-point is based on a possible biomass estimation error and will be explained a little later in both Section 3 and Section 4, including an exemplary calculation. In brief, the initial qs set-point was selected to lie in an area relevant for control purposes (reduced or negative Y L/G), in our case −10 and −15 p/cell*h.

## 3. Results and Discussion

### 3.1. MVDA for Assessing Lactate Metabolism

To capture the high dimensionality of a heterogeneous dataset, we employed PLS-R, a chemometrics tool [57,58] relatively straight-forward in its application [59]. The single most important feature we were interested in describing was the lactic acid metabolism. Therefore, only a handful of predictor variables were selected with the following considerations in mind:

1. We wanted to be able to set or influence the selected parameters easily, which is the case for the ones we selected, *i.e.*, pH.
2. We did not wish to become dependent on other parameters by including them in the analysis, which might then not be frequently analyzed, such as amino acids.
3. We did not want to include parameters or define new parameter ranges, which are fixed in a platform/manufacturing process, *i.e.*, pO_2_ or the pH (acid/base) regulation strategy.

From the available variables and the considerations above, the following were always available in all processes, for the whole process time, and therefore the most straightforward to describe lactic acid production or consumption (qLac): (i) glucose concentration (GLC); (ii) specific glucose consumption (qGlc); (iii) pH in the form of hydrogen ion concentration (H^+^); and (iv) lactic acid concentration (LAC).

**Figure 2:**
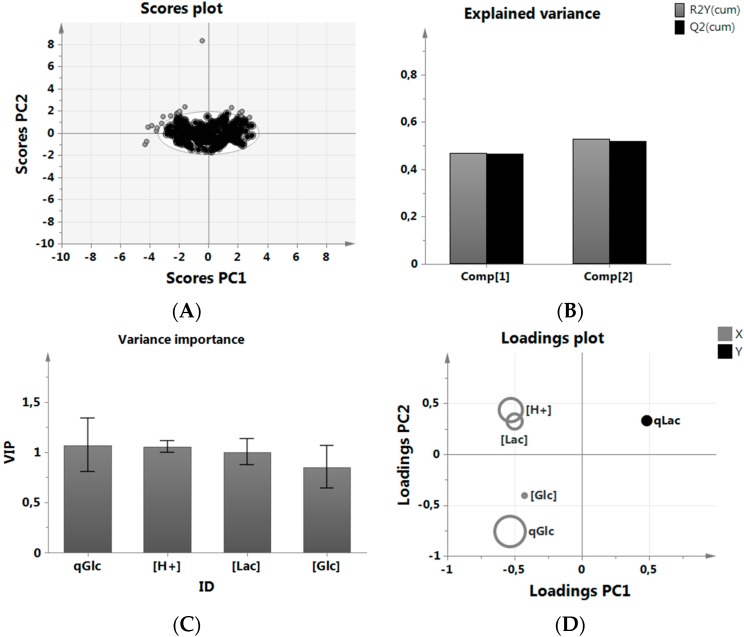
MVDA of the historical data set. (**A**) removal of outliers prior to analysis; (**B**) PLS-R model fit using two Principal Components, here Comp 1 and 2. R2Y(cum) indicates the explained variance, while Q2(cum) explains the predictive quality using one resp. two principal components; (**C**) VIP plot of the most important variables in the analysis sorted by relevance; (**D**) Loading scatter plot of data relationship between predictors (X) and predicted (Y) variable discriminated by VIP size.

A score scatter plot was used to identify all data inside a 95% Hotelling’s confidence ellipsoid and used in the subsequent analysis (Figure 2A). A new PLS-R model was built without the outliers, and variable variation (R^2^) and variable prediction (Q^2^) were calculated. We were interested in the ranking of parameters according to their relevance to reduce qLac to get a better understanding on what to focus on in our experiments. The resulting fit did not play a role in accepting or rejecting the predictive quality of the results as we did not use the model in a quantitative way (Figure 2B). High LAC as well as H^+^ prevented further increase of qLac [28], which could be mechanistically explained by chemical gradient action between the inside and outside of the cell [60,61]. The specific glucose consumption, meanwhile, was quite naturally indirectly proportional to lactic acid production and a much better predictor of metabolic behavior than, *i.e.*, GLC levels.

**Figure A1:**
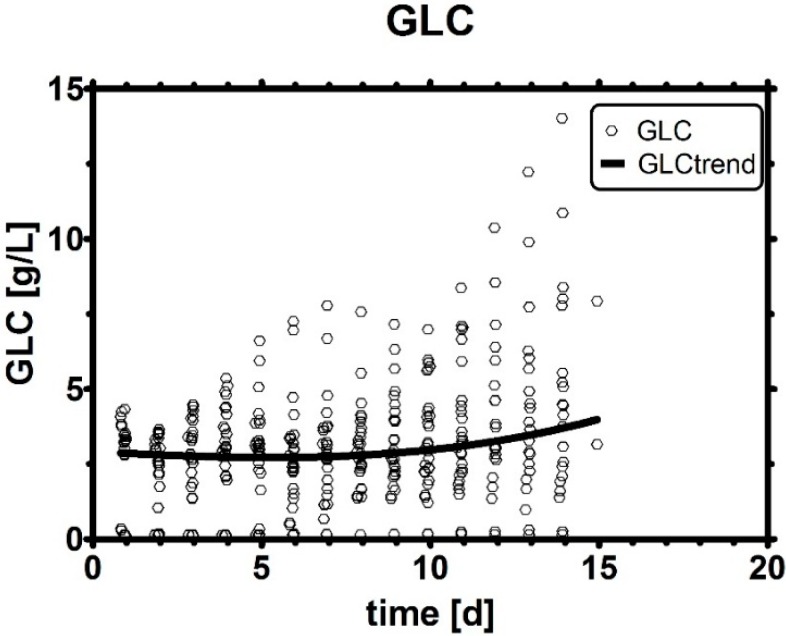
Historical GLC profiles. The GLC level itself was often inadequate to describe metabolic effects, such as the relation to qGlc in fed-batches. A robust third-order polynomial indicated an average trend of all runs, regardless of the individual trend in each cultivation.

**Figure A2:**
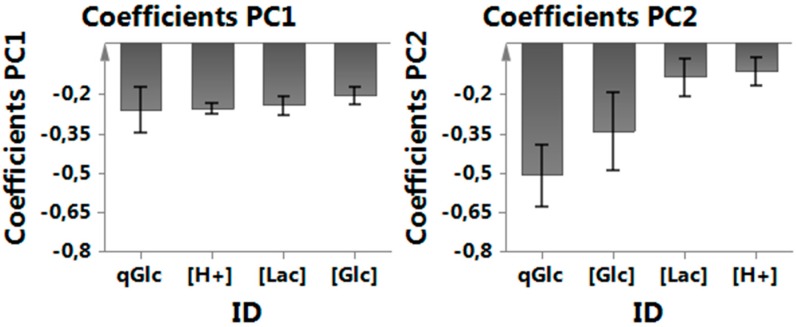
MVDA coefficients of PC1 and PC2 capturing direction of parameter effect on the target variable (qLac). PC1 captures most variance in the following order: qGlc, H+, Lac, GLC, while PC2 captures some remaining variance in qGlc and Glc but not Lac and H+ as they are already well represented by PC1.

A VIP (variance importance of the projection) analysis was used to simplify the multivariate data analysis by calculating a relative score for those predictor variables, which were used to describe the predicted variable qLac. A high VIP score (Figure 2C) resulted in a larger size representation of the given parameter in Figure 2D. The positions of predicted variable qLac together with the predictor variables explained the overall relationship of all parameters; H^+^ and LAC were clustered closely to each other and indicate a closer relationship, while all parameters, including GLC and qGlc, were positioned opposite of qLac, indicating an inverse relationship (see the Appendix for more information on the importance of GLC in this analysis, in particular Figure A1 and Figure A2). As GLC had the lowest VIP score, its importance may be understood to be low in this data set.

High GLC has an effect early in the culture while cells are “fit” to take up large amounts of glucose, so to speak. High GLC or overfeeding has almost no effect on cells later on—their maximum qs simply decreases over time, even if GLC was held perfectly constant over time. We can only speak for our experiments, but maybe this was already observed by other labs in which CHO cells were metabolically engineered to yield a reduced lactic acid profile. One would expect qLac to increase along with rising GLC levels, but from historical data this is not exactly the case, which implies that not GLC but the specific glucose consumption rate needs to be tightly controlled.

Summarizing our findings, we found that qGlc was by far the most important parameter to describe qLac behavior in process development runs, while H^+^ as well as LAC apparently played a role as well, although much less pronounced. GLC was statistically correlated with qLac due to the nature of the clone to consume lactic acid also during high glucose concentrations, but this particular finding was of little practical use. Therefore, the following experiments featured a modification of the most important parameters, which are qGlc and H^+^, in a glucose-limited state.

### 3.2. Lactic Acid Metabolism

**Figure 3:**
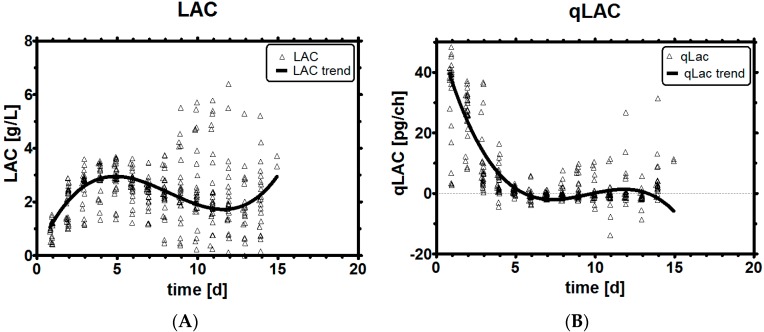
Historical LAC profiles (**A**); Historical qLac profiles (**B**).

The clone we investigated already featured a reduced LAC profile and was capable of lactic acid consumption as can be seen in Figure 3A,B. MVDA revealed that qLac can be expressed as a function of the parameters qGlc, LAC, H^+^, but not necessarily GLC; hence, the feeding had to be based on qGLC and not on GLC itself to improve lactic acid profiles, the latter being the normal control entity in industrial processes.

A strong early consumption of glucose is the main reason for high LAC levels later on, as will be seen a bit later in chapter 3.6. Historical process development data, where glucose flux was limited and low lactic acid levels were observed during the process, supports this statement. Le [4] shows that early intervention in the process affects the process outcome, which may be, for instance, the lactic acid profile. This intervention was demonstrated in this contribution with an early limitation of glucose availability, especially while the cells are still growing well. The benefit was to prevent LAC buildup and avoid many challenges with LAC and pH in cell culture. As any limitation of substrate may lead to reduced cell counts, we have tried both a weak and a strong limitation of qGlc to improve the process.

### 3.3. Impact of pH on qGlc

The historical processes all featured a pH set-point with an upper and lower range, inside of which no control agents are yet used—as result, the pH can assume several values, depending on the current control set-point. This raises the question if pH could be kept somehow stable by design and de-correlated from the typically concomitantly occurring undesired rise in LAC levels at alkaline pH. We differentiate between two different pH states: “pH low” (6.6–6.9) and “pH high” (6.9–7.2).

**Figure 4:**
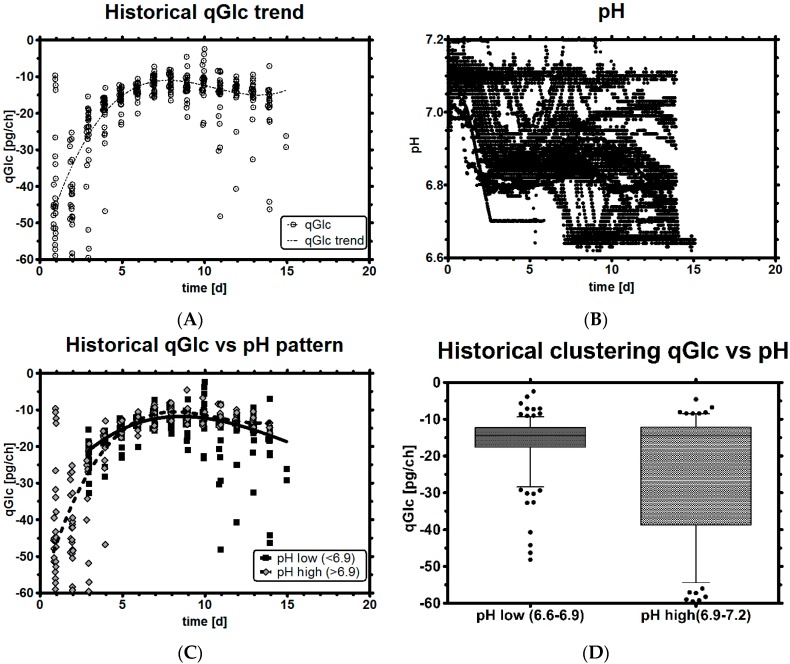
Historical qGlc profiles (**A**); On-line pH profile (**B**); Clustering of qGlc with a pH classifier (dark: low pH, bright: high pH) over time (**C**) and as box plot (**D**). A robust third-order polynomial function was applied in Graphpad Prism software to capture the general trend of a typical qGlc profile in (**A**) and qGlc in (**C**). No runs started with pH < 6.9 as pH initially falls and is then controlled.

Figure 4A,B show qGlc and on-line pH profiles of all 29 process development runs. The pH of all observations was split into two groups, higher or lower than pH 6.9, and plotted against qGlc. Under high pH conditions, a high qGLC range was observed, while at acidic pH, qGlc was more restricted (Figure 4C,D). As a result, high pH was often associated with high qGlc and *vice versa*. During the mid- and end-phase, pH did not impact or change qGlc much, while the effect that pH might exert on qGlc was strongest while the cells were growing well. Therefore, pH may be used indirectly to change qLac, as both qGlc and qLac are highly correlated (Figure 2D).

### 3.4. Set-Point Selection for qGlc

**Figure 5:**
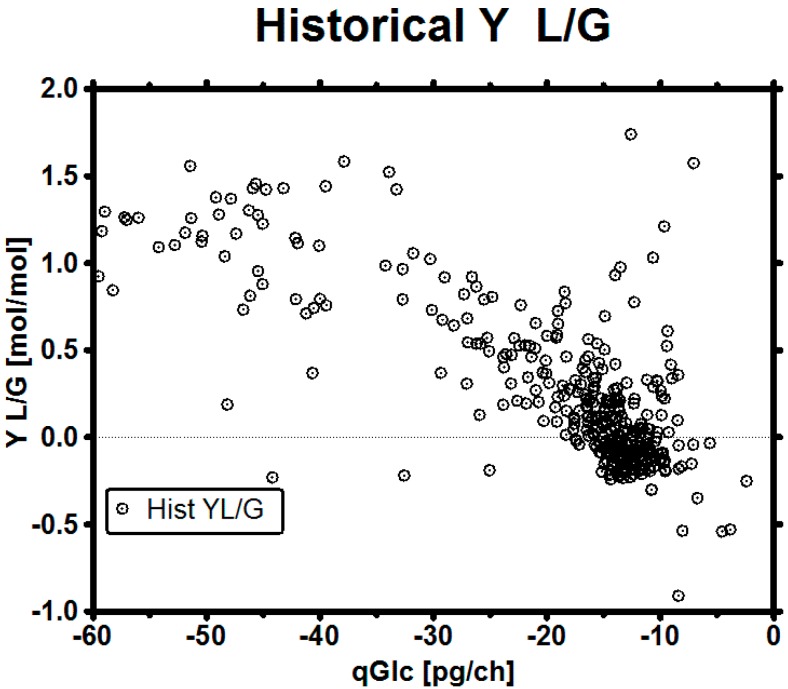
Historical yield qLac/qGlc (Y L/G) *versus* qGlc showing the consequence of particular qGlc on the yield.

The most important parameter which influences qLac and therefore LAC levels in the fermentation is qGlc. Other important parameters which also influence qLac, such as oxygen availability [62] or methods to improve pyruvate uptake into the tricarboxylic acid circle (TCA) [6], are acknowledged but out of scope of this publication. Historical data showed that the cells apparently never consumed less glucose than approximately 8 pg/ch (pg per cell per hour) and the gross of all measured qGlc lied in the range of 10–20 pg/ch. Plotting qGlc *versus* the yield qLac/qGlc (in short Y L/G) implies that, with the control of qGlc, a particular target yield might be achieved (Figure 5). This relationship may also be used to design a stoichiometric feed by trying to feed at a defined qGlc set-point in further experiments.

### 3.5. Impact of a Broad Range as qGlc Set-Point on the Lactic Acid Profile

First, we attempted to hold the cell’s qGlc set-point in a broad range between 10 and 30 pg/ch over the whole cultivation time by feeding at a target set-point rate which changes stoichiometrically with biomass. The feeding strategy is described in the experimental section (Table 2). For most of the time, the culture was controlled at a glucose level <0.36 g/L with a short excursion to up to 1 g/L when a high feeding set-point was automatically selected and the cells could not consume the substrate quickly enough before the next measurement.

**Figure 6:**
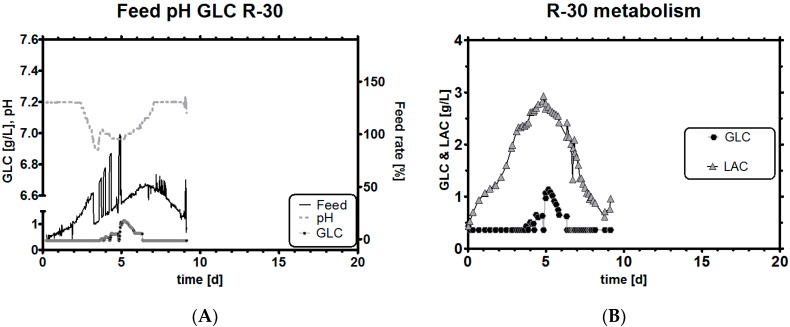

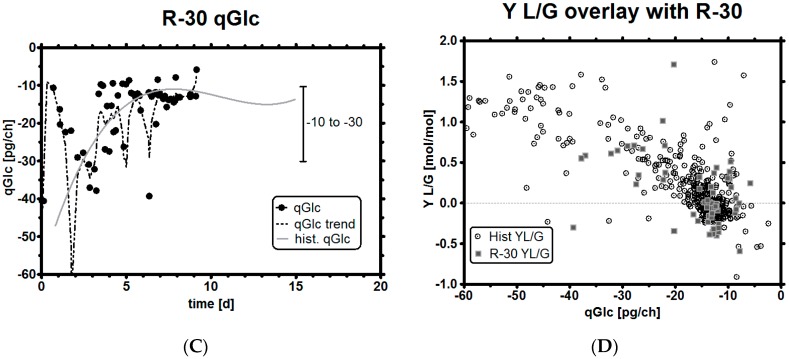
Experiment R-30, feeding profile with pH and GLC level (**A**); GLC and LAC concentrations (**B**); qGlc with trend and historical qGlc (**C**); overlay of resulting Y L/G profile with historical data (**D**).

In Figure 6A, it can be seen that the target specific rate of qGlc switched automatically between high and low set-point when certain conditions were fulfilled. Simply put, on-line pH was used as the main switch to affect the feed rate by changing the qGlc set-points between −10 and −30 pg/ch in real time. A second switch was the GLC level measured by an online analyzer, but pH had a higher hierarchy level as it was a faster available signal than the metabolite levels which were measured every couple of hours and so the associated events were rarely ever triggered. When the viable cell concentration reached a maximum, the high qGlc set-point was exchanged with the default set-point (10 resp. 15), because we know from experience that this clone does not require so much substrate so late in the cultivation.

Overall, this feeding strategy did not reduce LAC levels by much (Figure 6B) compared to historic reference runs (shown later). This was to be expected as we selected a purposely high qGlc set-point resulting in high glucose uptake rates. The qGlc trend of this run very much resembles the one from the historical runs which are overlaid in Figure 6C,D. As can be seen, qGlc falls well within the previously defined limits and shows the characteristics of a non-glucose-limited run (but *is* glucose- limited), which is relevant because it means two things: first, to achieve a lower qLac, a lower qGlc set-point must be selected (which could be relatively high so that the total carbon influx into TCA is not greatly reduced), and second, and perhaps more interestingly, a high GLC concentration is *not* necessarily required to keep the culture well. This is a very important statement because it implies that mammalian cell cultures may be operated in limitations if the biomass and qGlc can be estimated well. Both come with a particular error and, for this reason, a range rather than a fixed stoichiometric feeding point is necessary to keep the culture well supplied with enough nutrients over the whole process time. In the next chapter we briefly describe how a cell culture may be stoichiometrically fed if errors are rather low with experiment R-31.

### 3.6. Impact of a Tight Range as qGlc Set-Point on the Lactic Acid Profile

**Figure 7:**
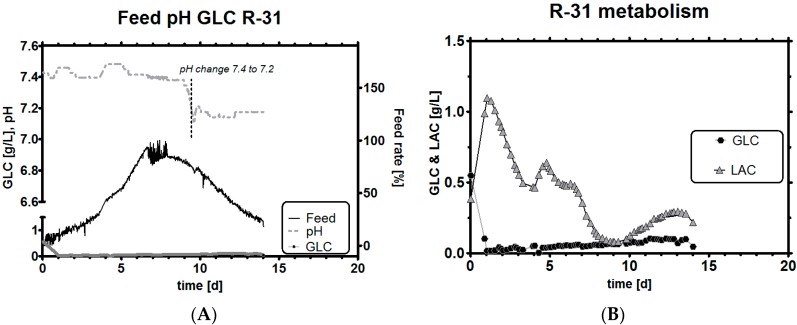

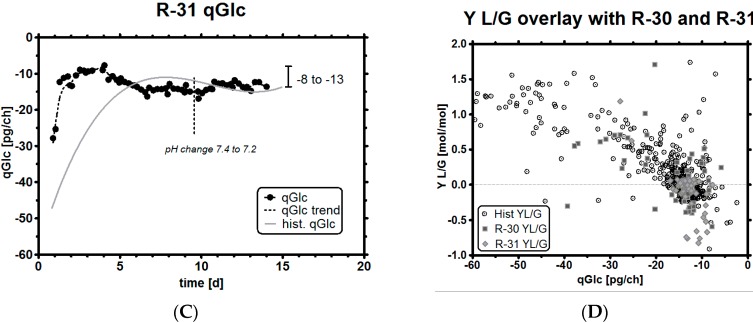
Experiment R-31, feeding profile with pH and GLC level (**A**); GLC and LAC concentrations (note that leftover GLC is consumed and results in a small LAC until it is consumed, but qs changes immediately to the target value once GLC is limited by the stoichiometric feeding strategy) (**B**); qGlc (including dotted trend line) and historical qGlc (full line) (**C**); overlay of resulting Y L/G profile with historical data (**D**). Both targets (qGlc and resulting lower Y L/G) can be better achieved due to the tighter control specification, when compared with run R-30. A comparison follows in Table 3.

We select a second experiment, in which we used a lower qGlc set-point criterion and selected a very stringent band between −8 and −13 pg/ch as the operational range. The rationale for such a strong glucose inhibition was that lactic acid production should, in theory, be very low because Y L/G approaches zero for this qGlc, as seen in Figure 5. The experiment R-31 shows a few noteworthy differences when compared to any historical process development runs, and, in fact, any runs we have found so far in the literature: first, pH was extremely stable (Figure 7A) during the whole process time and no control action by either acid or base was required. If, as a consequence of this strategy, pH should begin to rise too much in a run, acids are easier to add than to remove from the system. During the whole fermentation time, the tight qGlc set-point was never breached by more than 25% (which corresponds to the error of our biomass estimation method). Even when overestimating biomass, the consequence resulted in a total increase of qGlc by roughly 3 pg/ch, equaling roughly 16 pg/ch at the time of the largest error. This implies that even though biomass estimation is not perfectly accurate and deviates within a certain error range (as observed for the capacitance signal [63], but also any other signal), the qGlc set-point range simply needs to be selected high enough. A possible biomass underestimation cannot threaten the culture and if the set-point is still low enough, the benefits from a reduced LAC buildup can be still fully reaped (see Section 4 for an example).

**Table 3:** Statistical evaluation: in experiment R-30, a slight improvement of Y L/G and P/G compared to the historical runs can be seen. In experiment R-31, the improvements for Y L/G and P/G are most pronounced. The variation of qGlc could be massively reduced as can be seen in the low value for the standard deviation. N describes the number of available sampling events in the historical data and the experiments R-30 and R-31.

	qGlc [pg/ch]			Y L/G [-]			Y P/G [-]		
**ID**	**Historical**	**R-30**	**R-31**	**Historical**	**R-30**	**R-31**	**Historical**	**R-30**	**R-31**
**MEAN**	−22.39	−19.15	−13.92	0.41	0.18	0.03	0.29	0.31	0.36
**SD**	16.53	14.62	3.64	0.63	0.49	0.31	0.12	0.05	0.1
***N_All_ = 667, N_R-30_ = 60, N_R-31_ = 56***									

Another observation was the potential effect that pH may have had on qGlc. In all historical experiments, qLac was strongly correlated with pH, but here, for the first time, we could successfully decouple both parameters and investigate what happens if a high pH meets low LAC. During biomass overestimation, we could see that qGlc is higher than the historical reference but within the previously selected qGlc range of −8 to −13 pg/ch (Figure 7C). After the viable cell concentration maximum was reached, a pH change of −0.2 units brought the pH down from 7.4 to 7.2 (Figure 7A). After a delay of one to two days, qGlc decreased slightly by *ca.* 15%, which cannot be seriously discussed in terms of statistical significance. However, the direction of qs change after pH change would fit well into the overall picture of pH affecting qs (pH acidic: decreased GLC influx, pH basic: more GLC influx into the cells). It may be too early to conclude that pH could be used to modulate the maximum possible qGlc so extremely easily (which depends, among others, also on amino acid availability). However, the data suggest that exactly this may be the case, so this claim might be worth being explored by other research groups in the future.

We claim to have reached the goal of this study to reduce lactic acid profiles successfully, as can be seen in Figure 7B, with a minimum amount of experiments. Even though we exposed cell cultures to an unusually high pH of 7.4, which is usually correlated with high qLac, our feeding strategy showed exceptionally low LAC levels by controlling qGlc tightly and accurately. The culture could be kept in a metabolically highly interesting state for a duration of 14 days in a dynamic fed-batch process. The resulting Y L/G was, therefore, around zero most of the time and also assumed negative values, which are found to correlate with high titers in cell culture (Figure 7D) [5,64].

### 3.7. Adaptive Feeding Using Real-Time Switches

#### 3.7.1. Adaptive Feeding Using pH Correction

It is not in the nature of an error to announce an over- or under-estimation, so when exactly can the target set-point be switched on or off with an upper or lower range set-point, and how high or low can those possible set-points be defined? The answer to these questions lies in the enormous wealth of on-line signals which can be used exactly for this purpose [35,65]. In the first experiment, pH and the GLC level were used, and in another only pH was enough to switch the qGlc set-point to particular levels automatically. As the scale-down bioreactor model mirrors a manufacturing process with a floating pH dead band, we found pH suitable to automatically correct the feeding set-point. As a consequence, acid/base control for pH was not required at all. This may have had a positive impact on cellular physiology and, as a consequence, on the final titer. The strategy to include pH in set-point adaptation was adapted from Gagnon [42], but our control is more tightened due to the real-time and closed-loop approach instead of using predetermined rates. In the first experiment, we selected the switching criteria a little too broadly, and the set-point jumped at the smallest occasion, as can be seen in the feeding profile of run R-30 (Figure 6B). The reason for this was partially an unlucky first selection of the Boolean which often activated and deactivated the switch, but also the combined application of another switch from the online analyzer, as we had both signals at our disposal.

#### 3.7.2. Adaptive Feeding Using an Online Metabolic Analyzer

Ozturk described how online analyzers were used to hold a particular substrate or metabolite level [38]; however, for our problem statement this was not necessary, as the low glucose level posed no threat to the culture due to robust stoichiometric feeding. The online analyzer was working well but was actually not required to keep lactic acid levels low in a dynamic fed-batch. Therefore, we restricted the use of the set-point switch in experiment R-31 to pH only (Figure 7B). We conclude that one well-chosen switching criterion is enough for the task to keep the control strategy simple and intuitive. Although signals are available every minute, we advise to reduce control action to every half hour or more—otherwise, tiny excursions of the control range lead to feed corrections in the same time which makes the feeding profile impossible to interpret without strong smoothing. However, process development may very much benefit from highly frequently measured concentrations (*i.e.*, in the hour range) and unlock precise event detection, *i.e.*, after pulsing or shifting [66,67], to develop process understanding which may then translate into better process development and control.

### 3.8. Comparison of Experiments with Historical Performance

**Figure 8:**
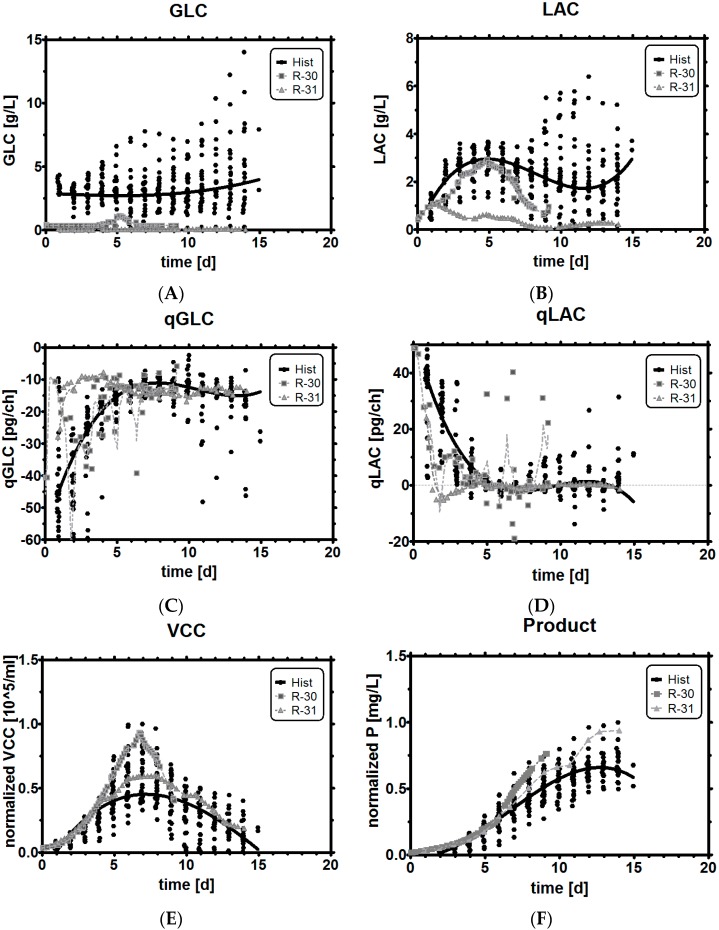
Benchmarking limited glucose control with historical data (black dots: historical data, squares: experiment R-30 with a broad qGlc set-point, triangles: experiment R-31 with a tight qGlc set-point. (**A**) and (**B**): metabolic profiles of glucose and lactic acid; (**C**) and (**D**): Specific glucose and lactic acid rates; (**E**) and (**F**): Viable cell concentration and product titer compared to historical data.

Direct comparison of key differences between historical data and the experiments reveals the following findings: Selecting the qGlc band for a stoichiometric feeding strategy in a very high range from −10 to −30 pg/ch led to comparable concentrations (Figure 8A) and metabolic profiles (Figure 8C) to the reference runs, even though glucose was limited at levels around 0.36 g/L (with a temporary shoot-up up to 1.2 g/L GLC). The operation in limited GLC conditions did not lead to a reduction of growth at all, as can be seen in the exceptionally high cell concentration (Figure 8E), but as cells started dying very fast in this process, a high final product titer was finally not obtained in this fermentation (Figure 8F).

**Figure A3:**
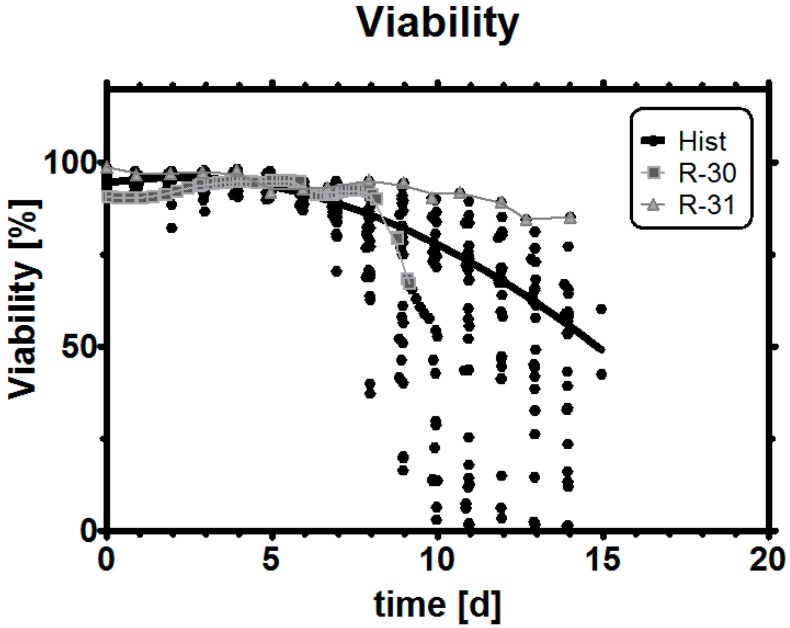
Viability trend in historical data, R-30 and R-31. The latter run had the highest viability at the process end. R-31 was tightly controlled throughout the process time in terms of metabolic state and featured a pH change from 7.4 to 7.2 between days 9 and 10.

In contrast to a very broad selection of qGlc, a very tightly controlled qGlc band between −8 to −13 pg/ch resulted in almost immediate LAC uptake from the environment (Figure 8B,D), while at the same time limiting the availability of GLC to a stoichiometric feed (Figure 8A,C). As can be seen, such a strong substrate limitation showed an effect on the growth profile, and on the maximum cell concentration (Figure 8E), which was somewhat low but still higher than the average mammalian cell cultivation. However, the declining phase was much softer and flatter, which may have been the reason for the high final titer at the end of the process (Figure 8E,F) as cells were still productive instead of being in the process of quickly dying. To be more precise, we observed that the majority of the cells in the tight qGlc limitation did not turn into dead cells, but lysed, which could be seen in the high viability (80%) at the end of the process run time (Figure A3 in the Appendix). This could indicate that cells took a different way to die than under regular process conditions. In a speculative afterthought, development of drugs which improve resistance to either cell lysis or apoptosis [68,69,70] might help to prolong cultivation (and production) time. A lower maximum viable cell count (VCC), but at the same time higher titer, suggests that the culture may have been simply more productive or productive for longer than the references.

From the experiments shown, we cannot truly distinguish which of the two parameters (qGlc or pH) finally led to more product. We assume that qGlc had the higher impact, simply because there is no truly direct mechanistic link between productivity and pH. It may have been a combination of both effects, but this statement would require much more rigorous testing, *i.e.*, by running a DoE to determine the exact contributions and interactions (of H+, qGlc set-point, qGlc upper and lower range, Y L/G, other low GLC levels, *etc.*), which was far out of scope of this contribution. Table 3 summarizes the experimental findings with regard to qGlc, Y L/G and Y P/G. The strongest limitation of average qGlc over the whole process time (−62% compared to the reference) resulted in a decrease of the Y L/G yield to almost zero, while at the same time the product yield was increased by 25%. For this we only needed to estimate biomass using a capacitance probe, which is already an accepted standard in many development labs. Usually, an increase in product yield has to be critically regarded, especially if the final titer turns out to be lower overall than the reference, but this is very clearly not the case here.

## 4. Conclusions

### 4.1. Eliminating the Root Cause for High Lactic Acid Concentrations

Historical data was analyzed by MVDA techniques where the specific glucose uptake rate was identified as the most relevant parameter to reduce the specific lactic acid production rate and, as a consequence, the lactic acid profiles of the culture. A feeding strategy was developed on this basis to set up a target specific glucose consumption rate for the whole process runtime of a fed-batch. This target rate was set up by estimating cell count via a capacitance probe, which can be used either for process monitoring [71,72,73,74] or control purposes [47,75]. Feeding was realized by supplying the cells stoichiometrically in a range between 8 to 30 pg/ch in real time with feed solution instead of using a previously defined off-line feeding profile which cannot react to deviations in the present process. Glucose is shown to be efficiently held constant to levels around 2–3 times the Km of mammalian cell lines during the whole process time without having a negative effect on viable cell concentration, as long as the specific glucose consumption feeding rate is selected to be high. Although the main effects leading to a reduction of lactic acid happen in the first days, it is well worth mentioning the remainder of the experiment. The proposed strategy also keeps the culture in the decline phase in a stoichiometrically fed state. This is a very important finding because it eliminates otherwise-required operator interventions and makes it easy to integrate it in an automatized manufacturing environment. We demonstrate that, if a strong limitation of the specific glucose consumption rate is selected, a very positive effect on the stability of the pH signal was observed together with a complete prevention of lactic acid build-up.

### 4.2. Directions from MVDA

Multivariate data analysis (MVDA) techniques can be tricky to use and sometimes misleading, as we have seen with the parameter GLC. The historical data set was comprised of glucose levels which are far above the cell’s physiological limit, where any effect on the specific glucose consumption rate could be expected. “Low” glucose levels (around 2–3 g/L) correlated to high lactic acid production rates, which makes no sense biochemically, but the largest part of the given data did not feature a glucose range in which qGlc was mechanistically affected. Because glucose concentration had the overall lowest weight in the analysis, and because the model was used in an explorative rather than quantitative way, the fit could be, from a statistical point of view, accepted. PLS-R was still very useful to correctly identify the exceptional importance of qGlc and the close relationship between H^+^ and LAC. Ranking their importance with regard to the impact on qLac could, furthermore, save valuable time by running the most interesting experiments first, instead of screening all eventualities. Conclusively, MVDA results were helpful to save time, but must be always reviewed carefully by considering whether the used data is really adequate and truly representative for the given problem.

### 4.3. Hypothesis for the Positive Effect of pH on Productivity

**Figure 9:**
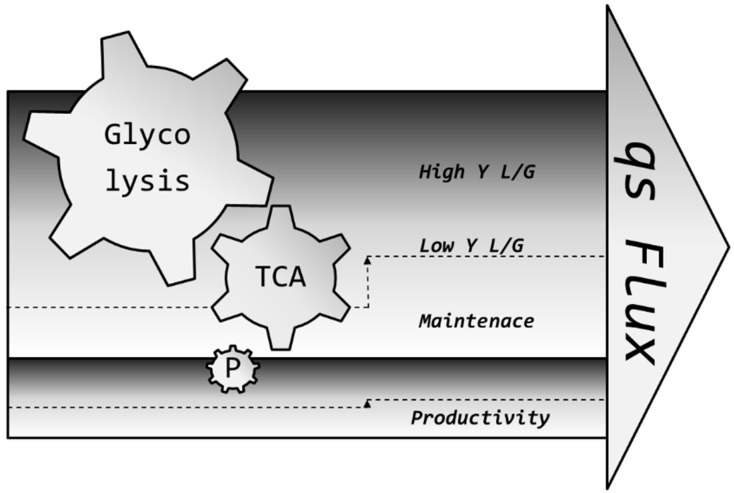
Suggested flux dependency on productivity. A higher overall flux may lead to a higher flux in all metabolic nodes, including lactic acid secretion and the one responsible for product formation. Usually, the basic consumption of cells does not change much because pH is controlled tightly. However, by setting and holding physico-chemical conditions which act on this flux by a high pH, while still operating in a relatively low Y L/G range, the overall higher flux may be cumulatively translated into higher productivity.

In our opinion, one facet of pH-dependent flux modulation remains overlooked until today and might be used to maximize the production of high-quality therapeutics of tomorrow: reverting the same method which researchers were using for years to reduce metabolic flux [30,76] into lactic acid and redirecting it to the TCA [50,77], we *increase* pH to maximize metabolism in substrate limitations. This is especially important in the light that lactic acid toxicity is actually more pronounced at lower pH than at higher pH [61,78,79,80] and may be the cause of more cell lysis [81], resulting in the lower productivity of a process. Flux increase is hypothesized to result from the cell’s regulation of its internal pH by a plethora of transporters [82,83,84,85] in the following manner: many enzymes involved in glycolysis are strongly affected by pH, among them those considered as metabolic bottlenecks, such as HK (hexokinase), PFK (phosphofructokinase) and others [86,87,88,89]. Even though the cytosol of the cell is strongly buffered, a certain gradient difference of around 1.5 units cannot be crossed if the cell should stay alive, not even if the cell in question is one of the strongest lactic acid producers [90] we are aware of today. Therefore, it is assumed that the internal pH, however buffered, must be affected by the external pH; thus, metabolism can be directed to different fates. The increase in flux is possibly distributed to all nodes and not just one node of the metabolism, among them the one responsible for product formation [77,91], as symbolized in Figure 9. We believe that the herein presented novel concept may lead to more biomass, which, in turn, may lead to higher titers [19], since more substrate leads to more cells which can produce more product due to an improved utilization of energy and nutrients.

### 4.4. Living with Uncertainty

**Figure 10:**
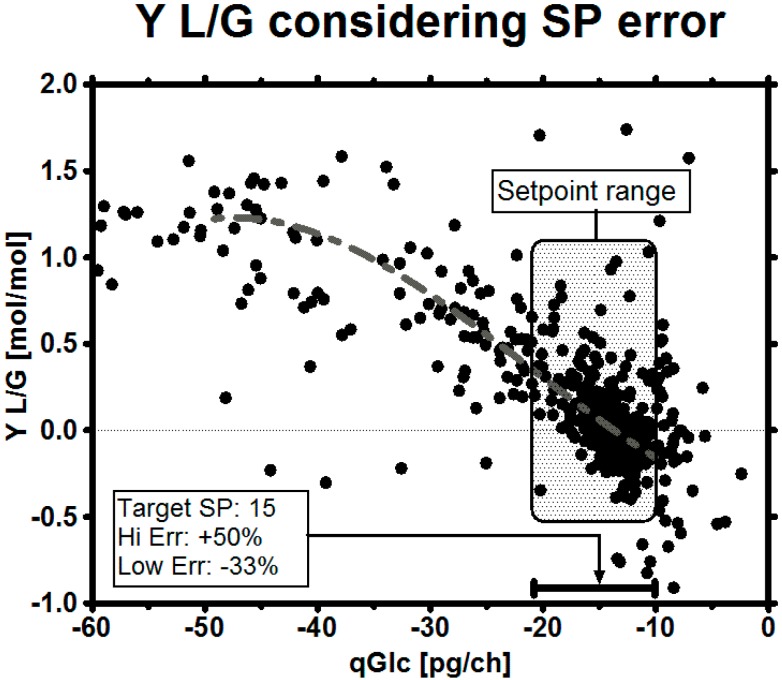
Metabolic control window with a suggested robust qGlc set-point. Data points represent both historical and experimental data. Depending on the task, the set-point range may be selected either conservatively (better overfeeding than starving, higher qGlc) or more courageously (improved metabolic state at the cost of reduced growth, lower qGlc). In general, the error of the online biomass estimation method may serve as a good first estimate for the range. It must be noted that the cells might not truly consume at a high qGlc just because it is desired by the operator and fed in this way, especially in the mid-phase of the culture—a fact which we suggest to solve by applying a high pH.

The biggest risk in the experiments was biomass underestimation, as this might mean starvation and an early stop of the process. In case that biomass estimation comes with a high error, two possibilities can be considered to cope with the uncertainty: changing the target qGlc set-point to a safer operation set-point or giving the target set-point a safe *range*, in which it can move and still satisfy the goals. The maximum error of biomass estimation may depend on the model by which it is assessed and have an asymmetrical distribution, *i.e.*, in an imaginary worst case scenario (Figure 10) of an overestimation of up to +50% and underestimation up to −33% (our biomass estimation in these experiments was in the range of maximum 25% over the whole process time, including the death phase of the culture). We want to point out that such a large uncertainty might not be as problematic as it sounds, as variation is a part of the daily business when dealing with biological systems [92]. In this contribution, we demonstrated how a robust qGlc set-point could be developed for any process using viable cell count estimation to calculate a safe feeding rate: in the historical data set we see that this clone almost always consumed at least 10 pg/ch to maintain metabolic integrity (Figure 7D). We exemplarily calculated a target set-point on the basis of the maximum error from biomass underestimation (Table 4) so, even with a high error on biomass, the culture will be supplied with sufficient nutrients in a glucose-limited fed-batch.

**Table 4:** Consequences of a high error for a robust set-point (SP) selection. To prevent starvation of the culture, the lower set-point range must be high enough to compensate for a possible underestimation of biomass.

**	*SP*	*Lower Range SP*	*Upper Range SP*
*Error*	0%	−33%	+50%
*Desired set-point considering error*	10	6.7	15
*Robust target set-point*	**14.9**	**10**	**22.4**

### 4.5. Limitations and Suggested Improvements

The range for qGlc enabled the introduction of a safety margin, which may be set up according to the error of the biomass estimation if certain conditions are fulfilled (*i.e.*, on-line available metabolic results *or* on-line pH). Especially in the case of a signal as simple as pH, of course not all is well. Especially in a buffered system, pH hardly changes due to cellular activity early in the process, but rather, because of process events, pH probes are known to drift. Therefore, other signals which may replace pH as the set-point switch are, for instance, capacitance, conductivity, pO_2_, pCO_2_, offgas, mass spectroscopy (MS), turbidity, base, inline microscopy, heat exchange, fluorescence, infrared, metabolic ratios, mass flow controller, online analyzer and many more [35,93]. If expensive high-tech equipment is not available, the controller output from proportional-integrative-derivative (PID) control [94,95,96] of suitable devices might pose an interesting alternative. A change in metabolic activity might be even better detected by using derivatives [2] or otherwise modified raw signals in combination with a robust signal-processing algorithm which may solve the possible problem with outliers very elegantly [55,97,98,99].

### 4.6. Summary and Outlook

The goal of the study was to reduce lactic acid accumulation in fed-batch fermentations in process development to increase overall process performance. We developed an adaptive feeding strategy which was based on real-time signals, capacitance and pH, and found that an on-line analyzer was not necessary for our intended control approach in glucose limitation. We have shown for the first time that the deliberate decrease of pH [26,29,32], which impairs lactic acid production but also cellular proliferation, was not necessary to stop lactic acid accumulation in the bioreactor, if the metabolic state could be controlled with a stoichiometric feeding strategy in which uncertainty is taken into account by design. As high lactic acid levels no longer posed a threat to the culture, a higher glucose consumption rate, favored, *i.e.*, by a high pH set-point, may boost productivity [81]. Experimental demonstration furthermore led to a more consistent pH and glucose profile, which might increase product consistency, as was reported by other authors [32,100,101], or simply process robustness. The uncertainty of biomass estimation was solved by designing a range of uncertainty-based feeding set-points, which may switch the feed rate in real time when combined with the previously suggested on-line-accessible parameters. The basis of our methodology, using specific uptake rates, is independent of scale, location and initial conditions. Therefore, the simplicity of the suggested method allowed a transfer of the desired *metabolic state* between piloting and manufacturing, which fits perfectly in the context of Process Analytical Technology (PAT) and Quality by Design (QbD) with respect to an improvement of robustness [1,102,103,104].

**Figure 11:**
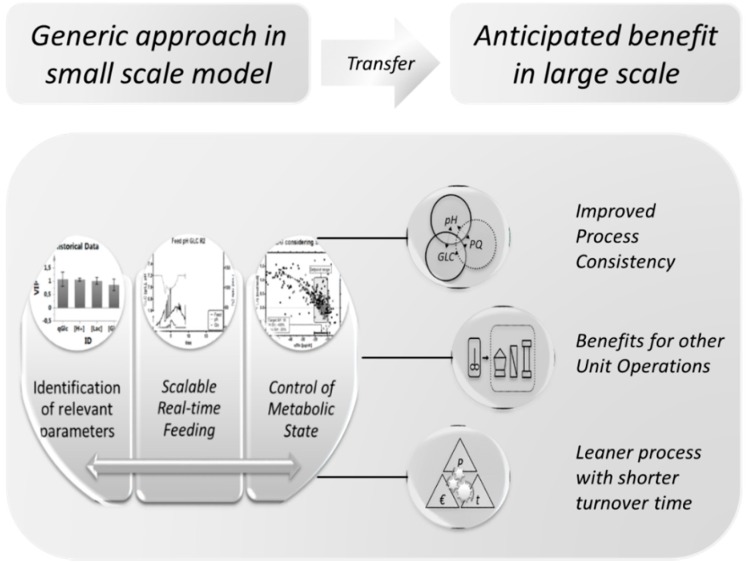
Generic approach for the holistic improvement during mammalian cell culture development.

A process run under such conditions from start to end holds a few yet unmentioned benefits in terms of cost of goods and productivity [105]: Healthy cultures with high viability are likely to create less cell debris because of the lower dead cell count, and the here-proposed feeding strategy is likely to result in a low residual substrate concentration at the end of the fermentation, which may potentially improve harvest efficiency. This naturally has an impact on subsequent unit operations [106], which, for example, include time-intensive separation, washing and purification steps [92,107] (Figure 11). We therefore hypothesize that the proposed strategy may be suitable to increase plant productivity and costs in the unit operations after the actual fermentation [108], which leads to leaner processes with a shorter turnover time and result in an overall improvement in productivity.

## Supplementary Files

Supplementary File 1

## Abbreviations

The following abbreviations are used in this manuscript:

CHO: Chinese Hamster Ovary
CHO-DG44: Clone B is derived from this cell line
Clone A: Clone in use for a biomass model based on capacitance
Clone B: Clone in this contribution (historical and experimental data)
CVRMSE: Coefficient of variation of RMSE (Root Mean Square Error)
DoE: Design of Experiments
Feed concentration, _Substrate_: Here: Glucose substrate concentration in the feed [mg/mL]
GLC: Glucose concentration [g/L]
GS-CHO: Glutamine-Synthetase CHO
H^+^: Hydrogen ion concentration [mol/L]
Hist: Historical runs from process development
HK: Hexokinase
IVCC: Integrated viable cell concentration [c/mL]
Km: Clone-dependent Monod constant for glucose affinity (0.1–1 g/L) [g/L]
LAC: Lactic acid concentration [g/L]
mAbs: Monoclonal Antibodies
MVDA: Multivariate Data Analysis
N: Number of observations
OD: Optical density [-]
PAT: Process Analytical Technology
pg/ch: Picogram per Cell per hour
PFK: 6-Phosphofructo-1-kinase
PLS: Partial Least Squares
PLS-R: Partial Least Squares Regression
Q^2^: Variable prediction coefficient
QbD: Quality by Design
qGlc: Specific glucose uptake rate [pg/ch]
qLac: Specific lactic acid uptake/production rate [pg/ch]
qOUR: Specific oxygen uptake rate [mmol/ch]
qs adapted: New qs set-point after online control action (*i.e.*, pH or online analyzer)
qs: General specific uptake rate notation, identical with qGlc as s stands for glucose [pg/ch]
R^2^: Regression Coefficient [-]
R-30: Experiment R-30, broad range for target qGlc set-point
R-31: Experiment R-31, tight range for target qGlc set-point
rlowess: Robust local regression using weighted linear least squares
SP: Set-point
SD: Standard Deviation
V_Reactorx_: Volume of the reactor [mL]
VCC: Viable Cell Concentration [cells/mL]
VIP: Variance importance of the projection, a measure of relevance of the parameter
Y GLC/OX: Yield glucose per oxygen [mol/mol]
Y L/G: Yield lactic acid per glucose [mol/mol]
Y P/G: Yield product per glucose [mg/g]
Y X/GLC: Yield biomass per glucose [cells/g]
Y X/GLN: Yield biomass per glutamine [cells/g]

## Appendix A. The Importance of Glucose

A high glucose concentration was not necessarily required to keep the culture well, as was shown in this contribution. Often, cell cultures in manufacturing and process development are operated “between 1 and 6 g/L” without further ado. One reason for this lies in the natural fear to lose a culture if the cells should be exposed to substrate limitations and starve, for instance because a stoichiometric feeding regime did not work as expected. Another reason for running at a high target glucose concentration range may have to do with glycosylation patterns of the final product which may be influenced by GLC levels [109]. However, the intracellular flux and concentrations might surpass the extracellular concentration of substrates in importance for the final structure of a complex protein [110]. Finally, some clones do not grow well below a certain glucose threshold and a limitation strategy is not the right approach for success; however, in this study, it worked very well.

To understand why GLC was often not a crucial parameter for lactic acid metabolism, it is necessary to take a look at the raw data. All historical runs started off with a remarkably high qGlc, which, over the course of a fed-batch fermentation, wore off and approached a certain plateau. In other words, qGlc (Figure 4A) was not solely governed by GLC as often assumed in microbial cultivations and appeared to be somewhat insensitive to a *high* GLC presence (see Figure A1) late in the culture, which means that even concentrated bolus shots would not change qGlc at this time point. GLC levels in cell culture were traditionally almost always operated way above the Monod constant Km, which depends on the genetic traits of the clone and may range between 0.1–1 g/L [111].

## Appendix B. Coefficients in Multivariate Data Analysis (MVDA)

The importance of parameters can be obtained by looking at the coefficients of the principal components, which also shows their effect on the target variable. (Here: qLac; positive coefficients indicate a direct correlation, while negative coefficients indicate inverse correlation. All coefficients were negative in this analysis). For their variable importance ranking, the coefficients are sorted regardless of their effect on the target parameter with decreasing importance, and this can be visualized by the VIP plot. In this dataset, the coefficients for glucose concentration (GLC) made apparently no sense. A negative coefficient would imply that a low glucose concentration was related to a high lactic acid production rate. GLC was almost always very high (>>>Km of glucose of the clone) and this clone was capable of consuming lactic acid even when there was plenty of glucose available. As a consequence, this behavior was captured in the statistical analysis and finally led to negative coefficients for glucose in this data set.

## Appendix C. Viability

R-31 shows very high viability of the remaining cell population at the end of 14 days, while at the same time the total viable cell count was decreasing. A large portion of cells were dying in a much more different way than in the other process development bioprocesses. Whether this should be traced back to the difference in pH or the metabolic state could not be investigated in more detail in this contribution.

## Notes

All figures were optimized for printing; however, they are also made available in color in the supplementary materials of this journal. We encourage the reader to take a look at them as tiny differences inside the paper become much clearer when looking at the figures in full scale at a high resolution.
